# Ameloblastin Inhibits Cranial Suture Closure by Modulating Msx2 Expression and Proliferation

**DOI:** 10.1371/journal.pone.0052800

**Published:** 2013-04-04

**Authors:** Phimon Atsawasuwan, Xuanyu Lu, Yoshihiro Ito, Youbin Zhang, Carla A. Evans, Xianghong Luan

**Affiliations:** 1 Brodie Laboratory for Craniofacial Genetics, Department of Oral Biology University of Illinois College of Dentistry, Chicago, Illinois, United States of America; 2 Department of Orthodontics University of Illinois College of Dentistry, Chicago, Illinois, United States of America; University of Texas Southwestern Medical Center, United States of America

## Abstract

Deformities of cranial sutures such as craniosynostosis and enlarged parietal foramina greatly impact human development and quality of life. Here we have examined the role of the extracellular matrix protein ameloblastin (Ambn), a recent addition to the family of non-collagenous extracellular bone matrix proteins, in craniofacial bone development and suture formation. Using RT-PCR, western blot and immunohistochemistry, Ambn was localized in mouse calvarial bone and adjacent condensed mesenchyme. Five-fold Ambn overexpression in a *K14*-driven transgenic mouse model resulted in delayed posterior frontal suture fusion and incomplete suture closure. Moreover, Ambn overexpressor skulls weighed 13.2% less, their interfrontal bones were 35.3% thinner, and the width between frontal bones plus interfrontal suture was 14.3% wider. Ambn overexpressing mice also featured reduced cell proliferation in suture blastemas and in mesenchymal cells from posterior frontal sutures. There was a more than 2-fold reduction of Msx2 in Ambn overexpressing calvariae and suture mesenchymal cells, and this effect was inversely proportionate to the level of Ambn overexpression in different cell lines. The reduction of Msx2 expression as a result of Ambn overexpression was further enhanced in the presence of the MEK/ERK pathway inhibitor O126. Finally, Ambn overexpression significantly reduced Msx2 down-stream target gene expression levels, including osteogenic transcription factors Runx2 and Osx, the bone matrix proteins Ibsp, ColI, Ocn and Opn, and the cell cycle-related gene CcnD1. Together, these data suggest that Ambn plays a crucial role in the regulation of cranial bone growth and suture closure via Msx 2 suppression and proliferation inhibition.

## Introduction

During skull development, calvarial bones expand from initial ossification centers toward the suture interface between adjacent bones [1]. Developmentally, this gradual intramembranous ossification is accomplished by the differentiation of ectomesenchymal cells into calvarial osteoblasts [2]. On a molecular level, membranous bone ossification is regulated by a number of growth and transcription factors as well as extracellular matrix proteins, including fibroblast growth factor receptors FGFR1 and FGFR2 [3], transcription factors CBFA1/RUNX2 [4]–[6], MSX2 [7], TWIST [8],[9], and matrix proteins osteopontin and tenascin [10],[11]. Abnormal changes in these factors not only affect bone thickness and mineralization, but also the expansion of bone along the ossification center/suture axis, resulting in either synostosis or open foramina.

From a clinical perspective, disturbances in normal skull ossification result in a number of pathologies with often dramatic consequences for children's health. Premature ossification of one or several sutures of the skull leads to an abnormal skull shape or retarded skull growth (Craniosynostosis, CS). The most common type of CS involves disturbances in the formation of a single suture (simple or non syndromic CS), and its etiology remains unexplained. Multiple suture synostoses (syndromic CS) are often associated with syndromes of genetic origin i.e. Apert syndrome, Crouzon syndrome, Pfeiffer syndrome and Saethre-Chotzen syndrome [12]. The incidence of non-syndromic CS in United States is about 34.3/100,000 live births while syndromic CS is rare; their incidence is about 1.5/100,000 [13]. The treatment of CS is composed of multiple surgical interventions to correct the abnormal shape of the head, give space for the brain to grow in a normal fashion and prevent the development of intracranial pressure leading to neurological and brain damage complications or death [14]. Complications from surgical treatment can lead to infection, re-ossification, and death from hemorrhage [15].

Cranial osteogenesis and the differentiated state of the bone/suture interface are regulated by extracellular matrix molecules that transmit signals across the cell membrane into the cytoplasm, activating a number of signaling pathways, which subsequently affect gene expression [16],[17]. One of the recently discovered members of the bone extracellular matrix is ameloblastin (AMBN), a glycoprotein previously only associated with the extracellular enamel matrix [18],[19]. Recent studies have noted Ambn expression also in dentin, cementum, pulp, cranial bones, and primary osteoblasts [20]–[25]. Moreover, Ambn has been shown to regulate Msx2 expression in ameloblasts [18] [26], and Msx2 mRNA expression was dysregulated in *Ambn* deficient mice [26]. In the present study, we hypothesized that Ambn might affect the growth of craniofacial bones and the patency of cranial sutures through its effect on Msx2 and possibly other mechanisms. Here we have tested this hypothesis using *Ambn* transgenic mice and *in vitro* models. Our studies reveal a distinct effect of *Ambn* on craniofacial bone growth and suture closure, and suggests possible mechanisms of action.

**Table 1:** Primer sequences for RT-PCR analysis.

AMBN	5′ aaaacccaccaacacctgag	3′ cattggtccccgagatattg
β-ACTIN	5′ gatcattgctcctcctgagc	3′ acatctgctggaaggtggac
IBSP	5′ gaagcaggtgcagaaggaac	3′ gaaacccgttcagaaggaca
CCND1	5′ cacacggactacaggggagt	3′ cgcggagtctgtagctctct
ColI A	5′ caccctcaagagcctgagtc	3′ tccgctcttccagtcagagt
MSX2	5′ agacatatgagccccaccac	3′ caaggctagaagctgggatg
OCN	5′ tcgtgtgtcttctccacagc	3′ tggccacttacccaaggtag
OPN	5′ ttccaaagagagccaggaga	3′ ttgtggctctgatgttccag
OSX	5′ cccctgttcttcacagcttc	3′ gggaaaacggcaaataggat
RunX2	5′ ccaccactcactaccacacg	3′ tatggagtgctgctggtctg

## Materials and Methods

### Transgene constructs and transgenic mice

Two transgenic constructs were generated using a modified pSKII-trans vector in which the *K14* promoter, the polyA signal (a generous gift from Dr. Elaine Fuchs, Rockefeller University), the *β-globulin* intron, the mouse *Ambn* coding region, or the *LacZ* gene was inserted. The *β-globulin* intron was used to ensure that the transgenes were transcripted properly. The transgenic fragments were freed from pSKII-K14-AMBN or pSKII-K14-LacZ by digesting the constructs with Sac I and Hind III, gel purified and microinjected into mouse zygotes [19]. Mice used in the present study included human keratin 14 (*K14*) promoter-driven *Ambn* transgenic mice (AmbnTg, E18.5, P1, 20, 35 and 60 days of age, *n* = 14) and *K14* promoter-driven *LacZ* transgenic mice (P1, *n* = 3). This study was carried out in strict accordance with the recommendations in the Guide for the Care and Use of Laboratory Animals of the National Institutes of Health. The protocol was approved by the Committee on the Ethics of Animal Experiments of the University of Illinois at Chicago (Permit Number: 11–077). For further analysis, mice were euthanized in a CO_2_ chamber followed by cervical dislocation, and calvarial bone and suture tissues were collected for fixation or RNA/protein extraction.

#### Genotyping

Genotyping was carried out using tails collected from AmbnTg or *LacZ* transgenic heterozygous litters [19]. The tails were lysed in DirectPCR (Tail) buffer (Viagen, Los Angeles, CA) at 50°C overnight and then 85°C for 40 min. PCR was performed using a set of specific primers for *K14* promoters: a forward primer 5′GCTTAGCCAGGGTGACAGAG 3′ and a reverse primer 5′CACAGAGGCGTAAATGCAGA3′. After 35 reaction cycles, aliquots of the PCR products were separated on a 2.0% TAE (Tris acetate EDTA) agarose gel, stained with ethidium bromide, and photographed under UV light [19].

### Dry skull preparation, whole mount X-gal staining and alcian blue/alizarin red staining

Dry skulls of three adult mice from wild-type and *Ambn* transgenic mice at postnatal day 60(n = 3) were prepared using *Dermestes* beetles. Briefly, the crude muscle and soft tissues were removed from the animal heads and left cool-dry for several days. The dry skulls were gently placed in a container with *Dermestes* beetles for several days at 30°C under close observation. Once the soft tissues of skulls were completely removed, the dry skulls were collected and cleaned meticulously with a soft brush. The dry skull, and especially the cranial suture of each animal was examined and photographed under a stereo microscope. Width of the posterior frontal suture of each animal was measured with a digital caliper and recorded in mm units. For whole mount X-gal staining, de-skinned animal heads were fixed with 4% paraformaldehyde in PBS at 4°C overnight. The samples were rinsed with PBS at room temperature 3 times for 15 minutes each and then incubated in the dark with a staining buffer containing 0.05 mM K_3_Fe(CN)_6_, 0.05 mM K_4_Fe(CN)_6_, 1 mM MgCl_2_ and 1 mg/ml X-gal at 37°C for 7 hours. For whole mount alcian blue/alizarin red staining, three embryos from the wild type and *Ambn* transgenic groups at embryonic day 18.5(n = 3) were fixed and dehydrated with 70%, 100% ethanol and acetone. The samples were stained with saturated alcian blue (Sigma, St Louis, MO) in 95% ethanol for 2 days, destained with 95% ethanol, and rehydrated. Subsequently, samples were then stained with saturated alizarin red S(Sigma) in 0.5% potassium hydroxide (KOH) solution for 2 days, destained in 0.5% KOH solution until all soft connective tissue turned clear and then stored in 80% glycerol.

### Tissue processing

Calvaria and cranial sutures from wildtype, *Ambn* or *LacZ* transgenic mice at age of embryonic 18 and postnatal 35 days (n = 3) were dissected and fixed with 10% formalin at 4°C and demineralized in a solution containing 45% EDTA, 4.5% NaOH and 1% formalin at 4°C until the demineralization was completed. The demineralized samples were embedded in paraffin, cut in 5 µm sagittal sections and mounted on glass slides. Thereafter, the samples were deparaffinized, rehydrated, and stained with Hematoxylin and Eosin or subjected to immunohistochemistry.

### Immunohistochemistry

Sections were deparaffinized, rehydrated, and treated with 6% peroxide and methanol followed by a brief incubation in 10 mM sodium citrate buffer with 0.05% Tween 20 at pH 6.0 for antigen retrieval. Sections were then incubated in 2% bovine serum albumin (BSA) for 30 minutes at room temperature to block nonspecific binding of the antibody. After blocking, sections were first incubated with affinity purified anti-Ambn antibody [19],[27] at dilution of 1∶200, and then with anti-rabbit secondary antibody (Abcam, Cambridge, MA) at dilution of 1∶2000. The expression of Ambn protein was visualized with a Histomouse Broad Spectrum AEC kit (Invitrogen, Carlsbad, CA) under a light microscope. As a negative control, non-immune rabbit serum was used instead of the primary antibody.

### BrdU and TUNEL staining

For BrdU staining, pregnant mice at E18 were injected with BrdU (100 mg/kg) intraperitoneally prior to euthanization. Calvaria and cranial sutures were dissected and processed for paraffin sections. The sections were deparaffinated, rehydrated, and stained with biotinylated anti-BrdU antibody. The BrdU positive cells were detected with the streptavidine-biotin system (Invitrogen). For TUNEL staining, sections were first exposed to proteinase K(20 µg/ml) at ambient temperature for 10 min, and then incubated with TdT staining solution at 37°C for 1 hour according to the manufacturer's instruction (Promega, Madison, WI). The fluorescence-dUTP-labeled DNA was visualized using a fluorescence microscope.

### Cell Culture

The mouse suture mesenchymal cells were isolated from the underlying dura mater and overlying pericranium of posterior frontal and sagittal sutures of wildtype and *Ambn* transgenic mice at postnatal 3 days, and maintained in α-minimum essential medium supplemented with 10% fetal bovine serum, 100 U/ml penicillin, 100 µg/ml streptomycin and 25 ng/ml Amphotericin B in a 5% CO_2_ atmosphere at 37°C. The medium was changed twice a week. To study the effects of Ambn on cell proliferation and differentiation, suture mesenchymal cells from wildtype and *Ambn* transgenic mice were subjected to regular culture medium or a mineralization induction medium containing 50 µg/ml ascorbic acid and 2 mM β-glycerophosphate, and cultured for 2–21 days. Upon terminating the culture, cells were used for MTT assay, alkaline phosphatase activity test, alizarin red staining, or RNA and protein preparation.

### Alkaline phosphatase and in vitro mineralization assay

After 7 days of culture in osteogenic media, cells were washed and stained with alkaline phosphatase substrate (Roche Diagnostic, Indianapolis, IN) to verify early osteogenic activity. After 21 days of culture in osteogenic media, the cells were fixed with methanol, stained with 10% alizarin red solution, and mineralized nodules were identified as red spots on the culture dish.

### Cell proliferation assay

Prior to termination of culture, cells were incubated in MTT solution (2 mg/ml of MTT in DMEM with 2% FBS) for 4 hours. To quantify proliferative activity, the MTT stained cells were lysed in HCL/Isopropanol, and the absorbance was detected at 570 nm with background subtraction at 630 nm [28].

### RNA extraction and RT-real time PCR

Total RNAs were isolated from mouse suture tissues or cultured cells using the TRIZOL LS Reagent (Invitrogen) according to the manufacturer's instructions. Two micrograms of total extracted RNA was applied toward cDNA generation with the Sprint RT Complete kit® (Clontech, Mountain View, CA). To quantify the mRNA expression levels of transcription factors and bone marker genes, real-time PCR primers were designed based on EMBL/GenBank searches (shown in Table I). Real-time PCR was performed using sequence specific sybergreen primers and the ABI Prism 7000 Sequence detection system (Applied Biosystems, Foster City, CA). Reaction conditions were as follows: 2 min at 50°C (one cycle), 10 min at 95°C (one cycle), and 15 sec at 95°C, and 1 min at 60°C (40 cycles). Samples were normalized using GAPDH. The analyses were performed in triplicate for three independent experiments to confirm reproducibility of the results. Relative expression levels were calculated using the 2^–ΔΔCt^ method [29], and values were graphed as the mean expression level ± standard deviation.

### Protein extraction and western blot analysis

Calvaria from 3 transgenic and 3 control animals at age P20 were collected and homogenized under liquid N_2_. Equal amounts of protein extracts in a lysis buffer containing 100 mM Tris HCl pH 9.0, 200 mM KCl, 25 mM EGTA, 36 mM MgCl_2,_ 2% deoxycholic acid and 10% DTT v/v were subjected to SDS–polyacrylamide gel electrophoresis (Biorad, Hercules, CA), and the separated proteins were transferred to a PVDF (Polyvinylidene Difluoride) membrane (Immobilon P®, Millipore, Billerica, MA). The membrane was incubated with rabbit anti-mouse Ambn [19],[27], Msx2, or CcnD1 antibodies (Abcam). Immune complexes were detected with goat anti-rabbit IgG horseradish peroxidase-conjugated secondary antibody (Molecular Probes®, Carlsbad, CA) and enhanced chemiluminescence reagents (Pierce Biotechnology, Rockford, IL). The amount of protein expression was compared after normalization with the amount of β-actin as an internal calibrator in each lane.

### Statistical analysis

Quantitative data were presented as means ± SD from three independent experiments and compared using Kruskall-Wallis one-way analysis of variance. The difference between groups was considered statistically significant at P<0.05.

## Results

### AMBN is expressed in calvarial bone

**Figure 1:**
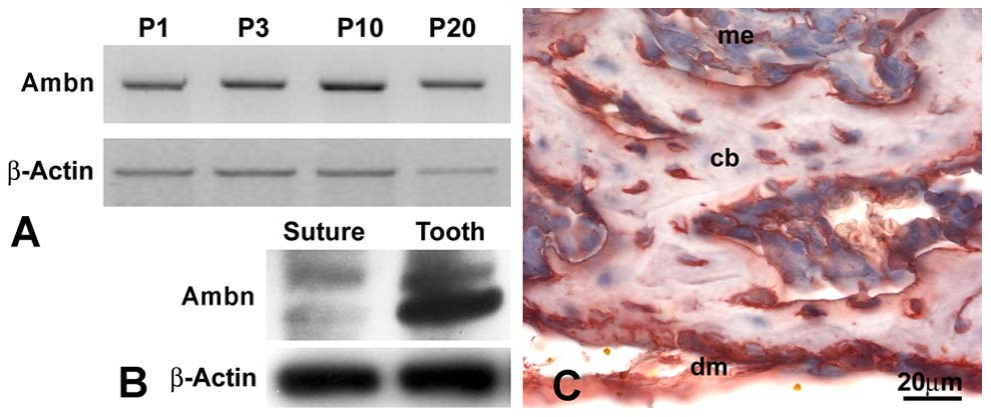
Ambn expression in cranial bone and dura mater. (A) RT-PCR analysis of Ambn mRNA expression in the posterior frontal suture region from 1, 3, 10, and 20 days postnatal WT mice. The β-Actin gene was used as internal control. (B) Western blot analysis of AMBN protein expression in postnatal day 3 posterior-frontal (PF) suture tissues and teeth. Two bands at 55 and 50 kDa were recognized. β-Actin was used as loading reference. (C) Immunostained sections of cranial bone and dura mater from 3 day postnatal wildtype (WT) mice demonstrated Ambn protein localization in calvarial bone (cb), dura mater (dm), and condensed mesenchymal cells (me). Note the high levels of Ambn protein in the dura mater and in calvarial osteoblasts. Bar = 20 µm.

Previous studies have demonstrated that the extracellular matrix protein Ambn is expressed in cranial tissues including enamel [30], cementoblasts [22], and cranial bones [24]. To examine the function of Ambn in cranial bone and suture formation, Ambn expression in mouse posterior frontal suture structures was characterized. RT-PCR analysis of samples ranging between postnatal days 1–20 revealed Ambn mRNA expression in postnatal cranial sutures (Fig. 1A). Western blotting recognized 50 and 55 kDa bands positive for Ambn in calvarial bone extracts (Fig. 1B). Using immunohistochemistry, Ambn protein was localized in calvarial bone, dura mater, and adjacent condensed mesenchyme (Fig. 1C).

### Five-fold AMBN overexpression in a K14-driven transgenic mouse model

**Figure 2:**
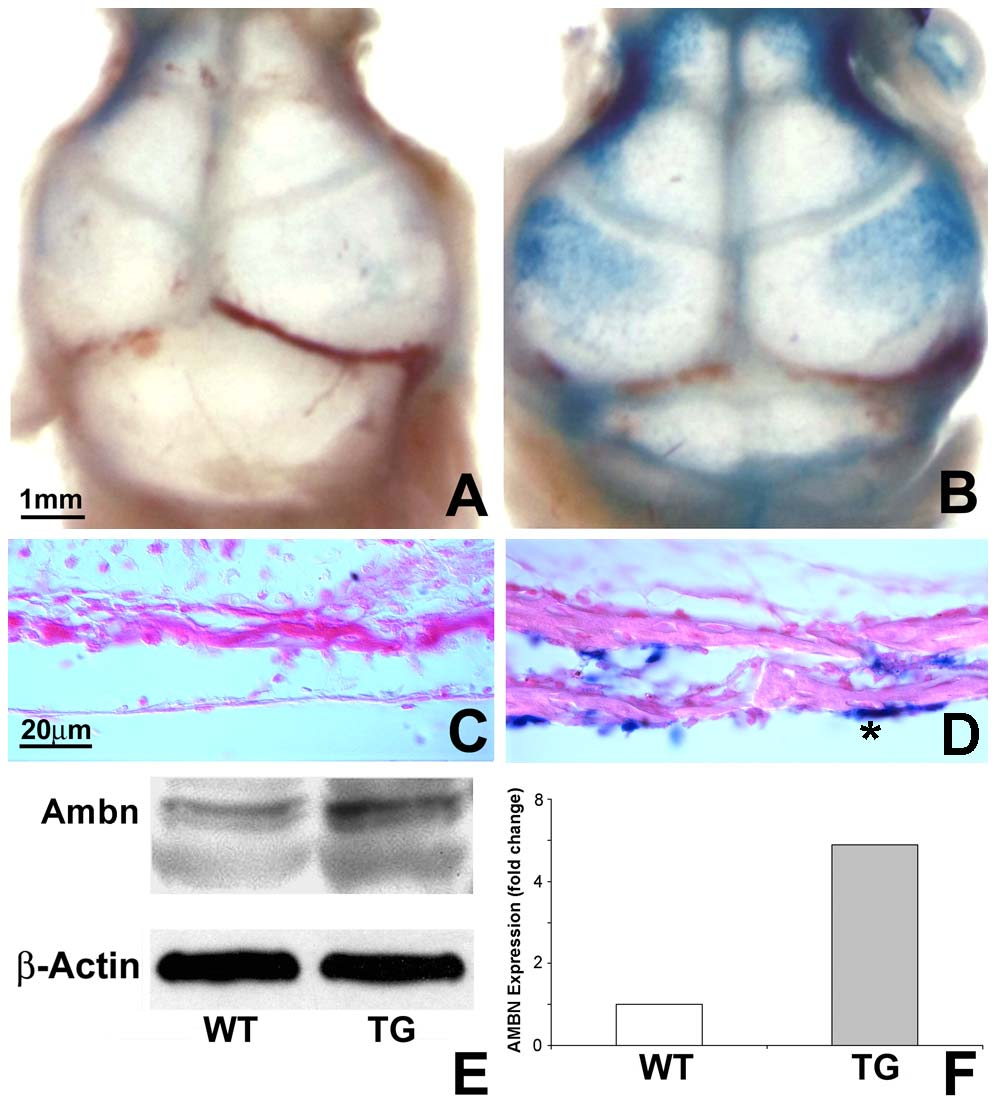
*LacZ* and *Ambn* transgene expression in cranial bone and sutures. (A and B) Whole mount β-gal staining of wildtype (WT) and *K14-LacZ* overexpressing mouse skulls. The β-gal blue signal was localized in cranial bones and sutures of the *Ambn* overexpressor. (C and D) Histological sections of β-gal stained and Eosin counterstained skulls were prepared from the coronal plane of the calvaria in the posterior-frontal region. Note the β-gal positive calvarial osteoblasts in the *Ambn* transgenic mouse sections. No blue staining of β-gal was detected in the WT skull section. Bar for A and B = 1 mm, C and D = 20 µm. (E) Western blot analysis of protein extracts from WT and *Ambn* transgenic calvarial bone and sutures at age postnatal day 3. The Ambn protein level in the transgenic mouse (right lane, upper panel) was 5-fold higher than that in the control mouse (left lane, upper panel) after normalization with β-actin (lower panel).

Gain of function mice are useful models to mimic syndromes of genetic origin such as suture synostoses (syndromic CS) [31]. We therefore generated *Ambn* transgenic mice using the human *K14* promoter to drive *Ambn* gene expression [19]. In addition, *K14- LacZ* transgenic mice were created to verify the location of the enforced *Ambn* gene expression [19]. Whole mount β-gal staining of the *K14*-driven *LacZ* transgenic skull revealed dark blue staining in parietal, frontal, maxillary, ethmoid and occipital bones and in calvarial sutures (Fig. 2B), when compared to wildtype mice (Fig. 2A). Histological analysis demonstrated β-gal blue staining in calvarial osteoblasts in transgenic mouse sections (Fig. 2D), but not in control mouse sections (Fig. 2C). When compared on western blots and after normalization with β-actin, the amount of Ambn protein from calvarial bones and posterior frontal (PF) sutures was 5 times higher in the *Ambn* transgenic mice than in wildtype controls (Figs. 2E and F).

### Posterior frontal suture fusion was delayed in Ambn transgenic mice

**Figure 3:**
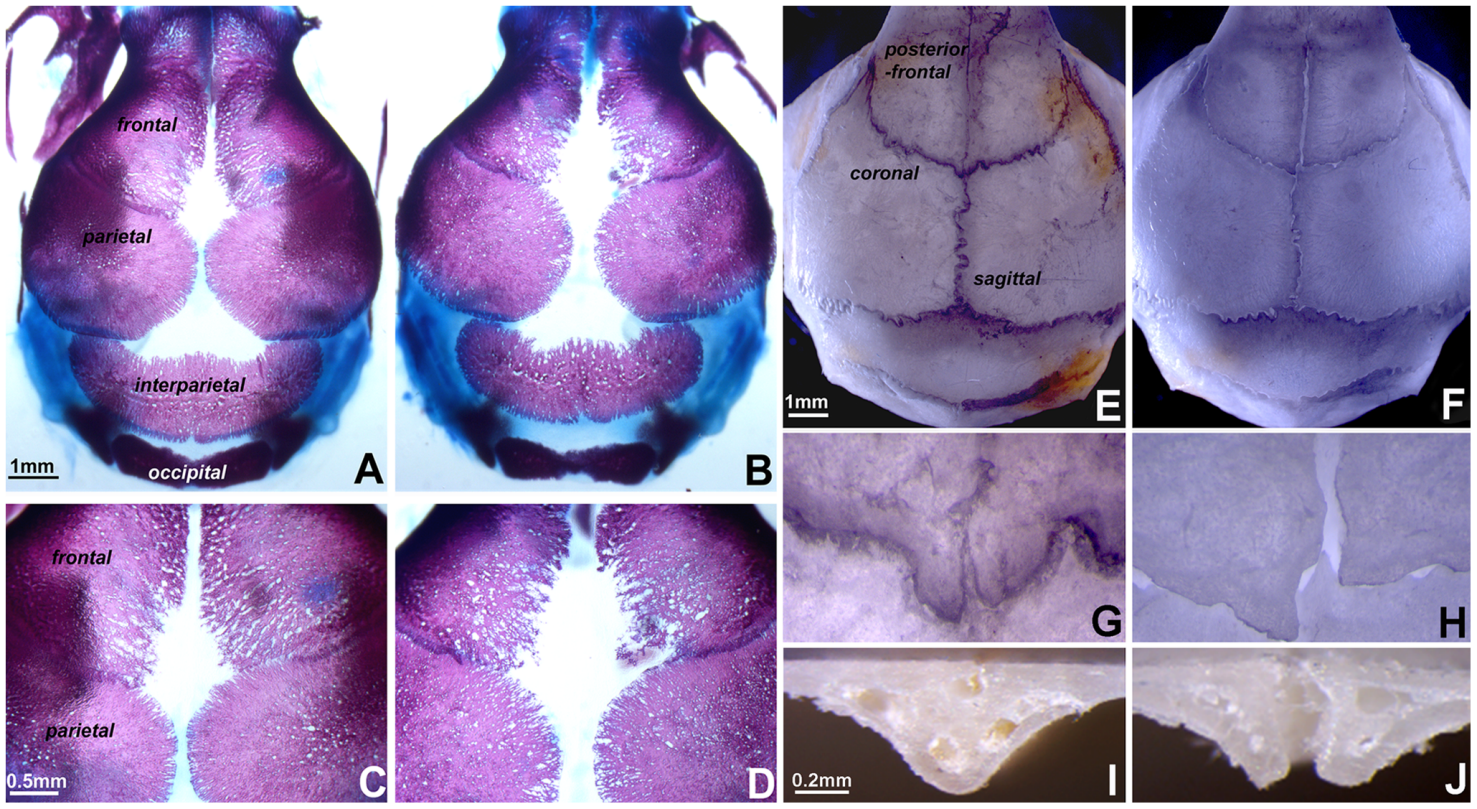
Delayed posterior frontal suture closure in *Ambn* overexpressing mice. (A and B) Whole mount alcian blue/alizarin red staining of wildtype (WT) (A) and *Ambn* transgenic (TG) (B) animals at embryonic stage day 18. The interfrontal gap was wider in the transgenic skulls compared to controls. (C and D) Higher magnification of the skulls shown in A and B. Note that the margins of the frontal bone osteogenic front were not well defined, and the interfrontal and parietal spaces were wider in transgenic mice compared to controls. (E and F) Dried skulls of WT and TG animals at 35 days postnatal. The PF suture was closed in WT mice (E), but remained patent in transgenic mice (F). (G and H) Higher magnification of the skulls shown in A and B. In TG mice, sutures remained open and adjacent bones overlapped (H). Cross-sections of the PF suture revealed the ossified areas of WT mice (I) and TG mice (J) at age 35 day postnatal. The PF suture was sealed by ossification in WT mice, while the left and right parietal bones were linked by soft tissue in TG mice.

To determine the effect of Ambn on cranial suture closure, skulls from *Ambn* transgenic and wildtype mice were compared using alcian blue/alizarin red staining for cartilage and bone mineralized tissue. *Ambn* transgenic mice at embryonic day 18.5 exhibited wider gaps of interfrontal and interparietal spaces (Figs. 3B and D) compared to those of their controls (Figs. 3A and C) (*n* = 3). There was less bone density and ossification along the suture (Fig. 3D), and the osteogenic margins of frontal bones were disorganized and not well defined (Fig. 3D), when compared to the wildtype control (Fig. 3C). This phenotype was indicative of a delayed development of the frontal bones. Skull samples of adult *Ambn* transgenic animals (age 60 days postnatal) revealed the patency of the posterior frontal sutures, revealing spanned gaps between the frontal bones (Figs. 3F and H), while the controls exhibited complete suture closure (Figs. 3E and G). The posterior frontal suture gap width in the *Ambn* transgenic mice was 0.12–0.42 µm (Fig. 3J), compared to 0.02–0.04 µm in controls (Fig. 3I) (*n* = 3).

### Ambn overexpressor skulls weighed less, and their interfrontal bones were thinner and wider

**Figure 4:**
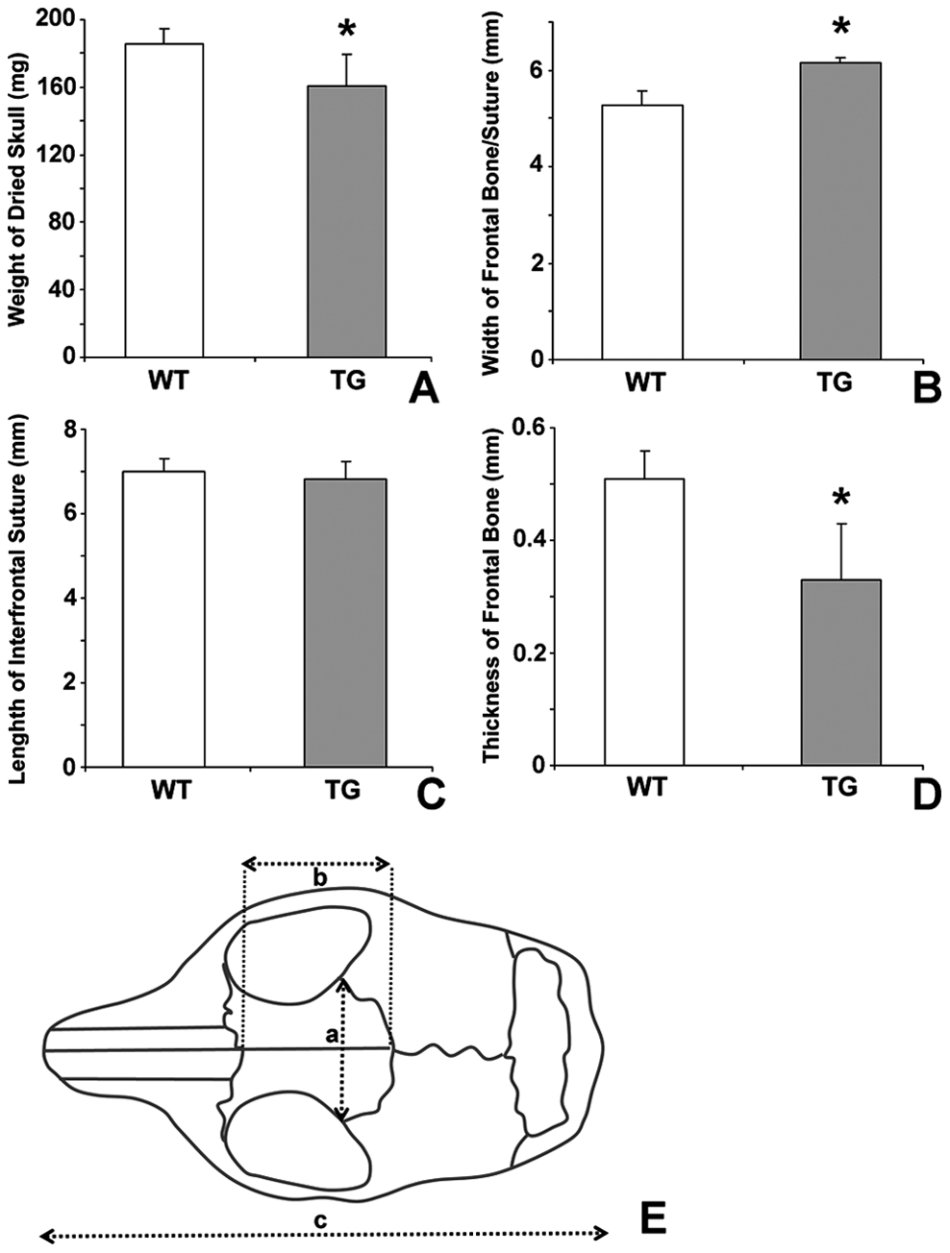
Weight and size comparison between wildtype and *Ambn* transgenic mice dried skulls. Dried skulls were prepared from 60 days postnatal wildtype (WT) and *Ambn* transgenic (TG) mice. Weight of dried skulls (A), width of frontal bone and suture (B), length of interfrontal suture (C) and thickness of frontal bone (D) were measured, and values were graphed as mean+/− standard deviation (SD). * represents significant difference, p<0.05 (Kruskall-Wallis one-way analysis). (E) Anatomical reference points for skull morphological measurements, including width of frontal bone and suture (a), length of interfrontal suture (b), and overall skull length (c).

To further characterize the phenotypes of *Ambn* transgenic mice, dried skull samples from wildtype and *Ambn* transgenic mice at age of postnatal day 60 were compared, resulting in at least three significant differences. First, there was a significant 13.2% reduction in weight of *Ambn* transgenic skulls compared to WT controls (Fig. 4A). Second, skull width of *Ambn* transgenic animals was 14.3% increased when measured at the interfrontal bone/coronal suture interface (Figs. 4B). Third, there was a significant 35.3% reduction in interfrontal bone thickness in transgenic mice (Fig. 4D). Interestingly, there was no significant difference in the length of skulls (data not shown), and in the length of the posterior frontal suture, when comparing wildtype and transgenic groups (Fig. 4C).

### Incomplete posterior frontal suture closure and reduced proliferation rates in Ambn transgenic mice

**Figure 5:**
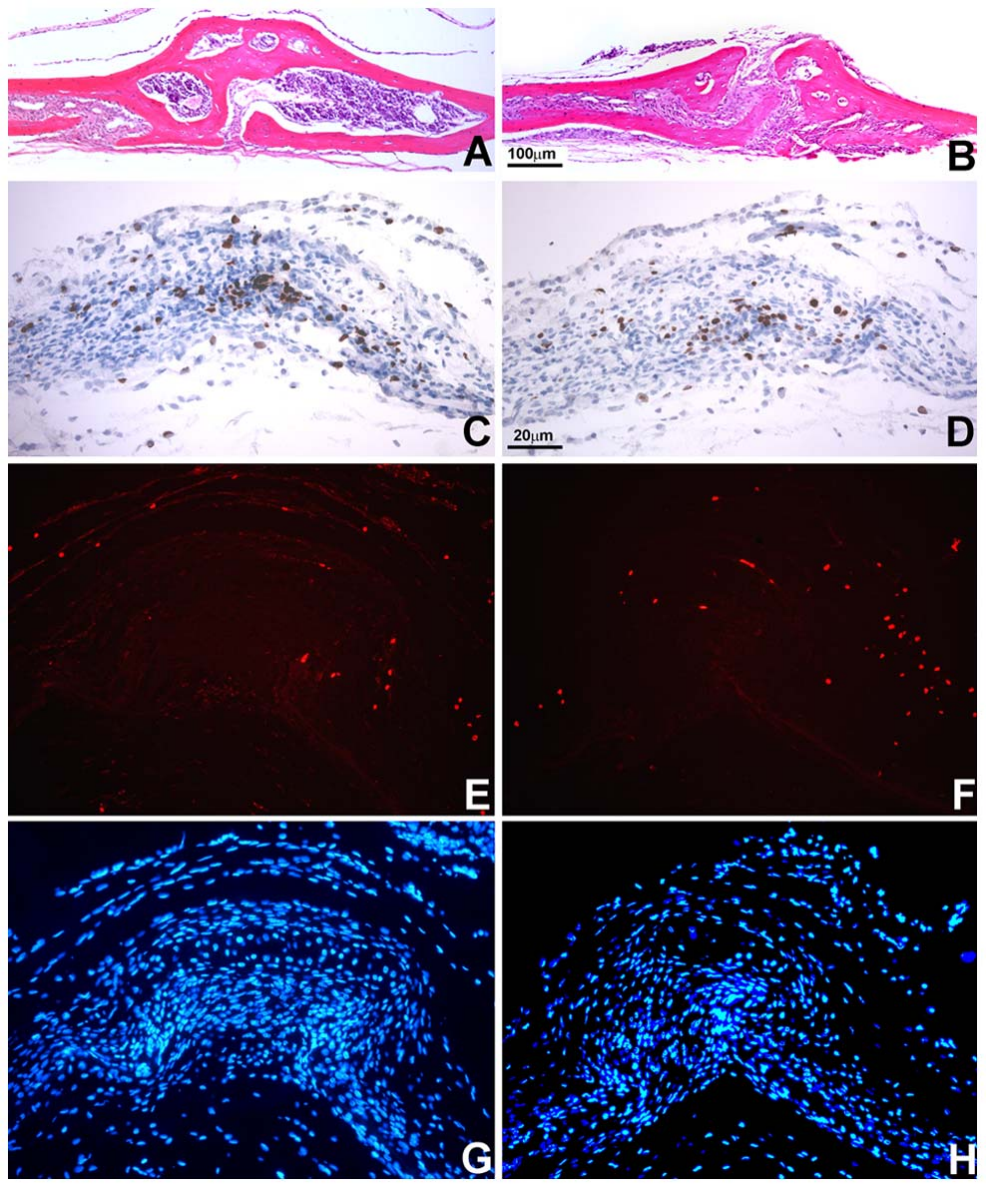
Role of Ambn in suture development. (A and B) Cross-sections of the PF suture revealed the ossified areas of wildtype (WT) and Ambn transgenic (TG) mice at age of 35 days postnatal. The PF suture was fused by ossification in WT mice (A), while the left and right parietal bones were interrupted by soft tissue in TG mice (B). (C and D) BrdU labeling of proliferative cells in developing sutures. Note the accumulation of BrdU-labeled cells in the suture center of WT mice (C) compared to TG mice (D). (E and F) TUNEL staining in developing sutures. Note the red labeling for TUNEL-positive cells in WT and TG mice. (G and H) DAPI counterstaining of (E and F).

Histomorphological analysis demonstrated complete closure of the posterior frontal suture in control mice (Fig. 5A) in contrast to incomplete closure in transgenic mice (Fig. 5B). The micrograph was a fused bony bridge formation on the telencephalic aspect of the calvaria in control mice (Fig. 5A), while the frontal bone arch was interrupted by a soft tissue interface in the *Ambn* transgenic mice (Fig. 5B). The interrupting soft tissue extended from the periosteum to the dura mater in vertical direction (Fig. 5B). BrdU staining of the developing posterior frontal suture (E18) revealed 29% less proliferating cells in *Ambn* transgenic mice. The number of BrdU positive cells per 0.01 mm^2^ in the control group was 124+/−5.6 while the number in *Ambn* TG was 88+/−2.8. In contrast, the number of TUNEL positive cells was 1.3-fold higher in *Ambn* overexpressors (Figs. 5D and F) when compared to wildtype mice (Figs. 5C and E).

### Ambn inhibited mouse suture mesenchymal cell proliferation and differentiation in vitro

**Figure 6:**
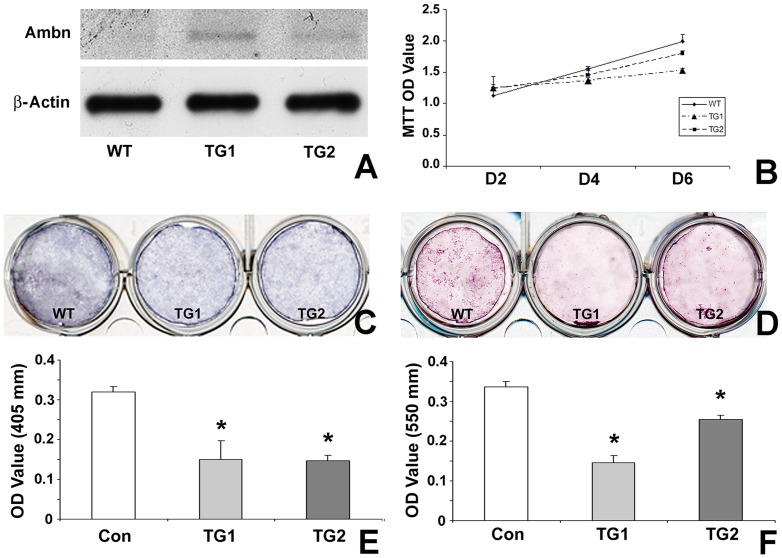
Effect of Ambn on proliferation and differentiation of suture mesenchymal cells. In this study, mesenchymal cells from wildtype (WT) mice and *Ambn* overexpressor (TG) sutures were cultured in osteogenic induction medium. (A) Ambn protein expression in suture-derived cells. β-Actin was used as loading control. (B) MTT assay to compare cell proliferation in WT and TG mesenchymal suture cells. The measured absorbance (mean+/− SD) is proportionate to the number of living cells. (C) Alkaline phosphotase activity staining at day 6 of culture identified differentiated cells positive for alkaline phosphatase with blue staining. (D) Alizarin red staining for mineral nodule formation. Cells were cultured for 21 days in osteogenic induction medium. Mineral nodules were stained in red. All experiments were carried out in triplicate and repeated three times. * represents significant difference, p<0.05 (Kruskall-Wallis one-way analysis).

**Figure 7:**
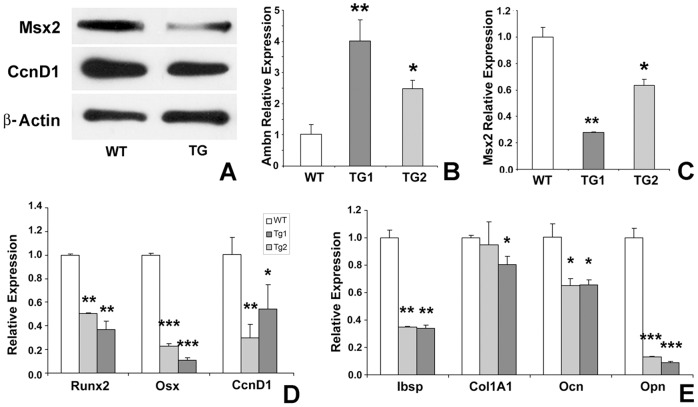
Expression of Msx2 and downstream genes in suture and suture-derived mesenchymal cells of wildtype and *Ambn* transgenic mice. (A) Western blot analysis of Msx2 and CcnD1 expression in sutures. Suture extracts from wildtype (WT) and *Ambn* transgenic (TG) mice were processed for immunoblotting with antibodies against Msx2 and CcnD1. β-Actin was detected as a loading control. (B and C) Quantitative RT real time PCR analysis of Ambn (B) and Msx2 (C) gene expression in suture-derived mesenchymal cells. (D and E) Quantitative RT real time PCR for Msx2 downstream target genes in cultured mesenchymal suture cells from WT and TG mice; (E) Runx2, Osterix (Osx), and Cyclin D1 (CcnD1) transcription factors; and (F) Bone sialoprotein (Ibsp), Collagen I (Col1A1), Osteocalcin (Ocn), and Osteopontin (Opn) matrix proteins. Expression levels were calculated relative to GAPDH gene expression levels using the 2^–ΔΔCt^ method, and values were graphed as the mean expression level ± standard deviation. *,**, and *** represent significant difference, p<0.05, p<0.01, and p<0.001 respectively (Kruskall-Wallis one-way analysis).

To determine how Ambn affects suture development, suture mesenchymal cells were isolated from underlying dura mater and overlying pericranium of posterior-frontal sutures of wildtype and transgenic mice. Suture mesenchymal cells from wildtype mice expressed basal Ambn protein levels (Fig. 6A), while the cells from two different transgenic mice over-expressed Ambn protein at about 4.05(TG1) and 2.5(TG2) times higher levels (Figs. 6A and 7B). To further examine whether Ambn expression levels affect cell function, proliferation of suture mesenchymal cells was analyzed using the MTT assay. There was an inverse relationship between Ambn expression levels and cell proliferation capacity, with highest MTT OD value in wildtype cells, followed by TG2 and then TG1 cells (Fig. 6B). In addition, there was a substantial reduction in alkaline phosphatase activity and Alizarin red staining in the two cell lines from Ambn overexpressor mice, TG1 and TG2 (Figs. 6C and D). Remarkably, the high Ambn overexpressing cell line TG1 had a stronger effect on Alizarin red-detected mineralization inhibition than the less Ambn overexpressing cell line TG2, suggesting that Ambn over-expression inhibited suture mesenchyme osteogenic cell differentiation in a dose-dependent manner (Figs. 6D).

### Ambn regulated Msx2 and downstream gene expression

Ambn overexpressor mice displayed posterior frontal suture patency, a phenotype previously reported in *Msx2* deficient mice [32]. In addition, Ambn over-expressing suture mesenchymal cells displayed lower proliferative capacity and delayed differentiation similar to the effects of *Msx2* deficiency on mesenchymal cell function [32]. Therefore, Msx2 expression in suture tissues as well as suture mesenchymal cells from *Ambn* transgenic and wildtype mice was examined. Western blot analysis revealed that Msx2 and CcnD1 expression in calvarial bones and PF suture tissues of *Ambn* transgenic mice were downregulated (Fig. 7A). Msx2 mRNA expression in suture mesenchymal cells from wildtype and transgenic mice was reverse proportional to Ambn mRNA expression, when comparing cells from two different transgenic lines (Fig. 7B and C). To determine whether Ambn affects expression of Msx2 down-stream target genes, expression levels of osteogenic transcription factors Runx2 and Osx, bone matrix proteins Ibsp, ColI, Ocn and Opn, as well as the cell cycle-related gene CcnD1 were detected using quantitative RT real time PCR. Our data demonstrated that Ambn overexpression resulted in significant decreases in bone transcription factor and bone mineralization marker gene expression in a dose-dependent manner (Figs. 7D and E). Similarly, CcnD1 expression was downregulated in TG1 and TG2 cells. Together, these studies indicate that Ambn inhibits cell proliferation and reduces Msx2 and downstream gene expression *in vivo* and *in vitro*.

## Discussion

Transgenic animal models are ideal biological systems to test the effect of forced gene expression on the development of a phenotype. In the present study we have used the human *Keratin 14* promoter to overexpress the extracellular matrix protein Ambn in calvarial osteoblasts and suture mesenchymal cells. Keratin 14(K14, Cytokeratin 14 =  CK14) is commonly known as an epithelial protein encoded by the *KRT14* gene. However, K14 expression in calvarial osteoblasts has been previously reported and confirmed by Western blot [33]. In addition, reports of keratins in non-epithelial cell lineages have become more frequent in the literature, including mouse studies related to keratins involved in endodermal and mesoderm adhesion in early embryogenesis [34] (Vijayaraj et al. 2010, reports on K19 in dental papilla and dental pulp [35] (Webb et al. 1995), microarray and western blot detection of K18 in cementoblasts [36] (Dangaria et al. 2011), findings of K19 and K8 in mature striated muscle [37] (Shah et al. 2012), and K8 and K18 expression in mesenchymal progenitors of regenerating limbs [38] (Cocoran and Ferretti 1997). Here, *K14* expression in calvarial osteoblasts was further confirmed by β-gal staining for the *K14 LacZ* transgene in *K14 LacZ* transgenic mice using both histology and whole mount staining. The *K14* promoter system has become a promoter of choice in our and other laboratories because of its efficient expression of many transgenes [39],[40]. *K14*-driven overexpression of Ambn in mouse calvaria resulted in a robust 5-fold enhancement of Ambn levels in calvarial tissue extracts.

Our study indicates that Ambn was expressed in developing calvarial bone and sutures. This finding matches other reports of Ambn expression during bone formation [25],[41] and expands its original concept as an ameloblast-specific gene product [42]. However, when compared to the developing enamel matrix, expression levels were less, suggesting that Ambn may not act directly on bone development by controlling crystal growth as it does in the enamel matrix [29],[43], but rather indirectly as a signaling molecule affecting the expression of transcription factors and extracellular matrix signaling pathways [23],[44],[45]. This concept is supported by findings reported in the present study that already small differences in *Ambn* concentration have significant effects on cell behavior, including cell proliferation and mineralization.

Our human *K14*-driven *Ambn* overexpressor mice suffered from delayed and/or incomplete cranial suture closure and displayed patency of the posterior frontal suture. Moreover, frontal bones of *Ambn* overexpressors weighed less and were thinner, suggestive that Ambn was involved in tissue growth or mineralization or both. We interpret these findings to indicate that Ambn plays an important role in the growth and development of cranial bones and subsequent suture closure. Our concept that *Ambn* affects mineralized tissues outside of teeth is supported by earlier studies related to the effect of *Ambn* on bone [41]. In our study, the phenotype of incomplete suture closure in *Ambn* overexpressing mice is explained by two related *in vitro* findings, namely (i) a significant reduction in suture mesenchymal cell proliferation and (ii) a downregulation of the cell proliferation marker cyclin D1 (CcnD1). In addition, studies in our and other laboratories have also provided evidence for the inhibitory effect of Ambn on cell proliferation, e.g. an increase of the cell proliferation inhibitors p21 and p27 in Ambn overexpressing ameloblasts [26],[46]. In contrast, there have also been reports suggesting that Ambn promotes bone healing by enhancing progenitor cell proliferation [41] (Tamburstuen et al. 2011) and regulates osteogenic differentiation [47] (Iizuku et al.). We propose that such findings may be due to different concentrations of Ambn acting either as a signaling molecule or a structural matrix protein [48] (Diekwisch 2011).

Another factor responsible for the inhibition of suture closure by Ambn overexpression is the transcription factor Msx2, a causative gene involved in human cranial suture pathologies such as CS and EPF [49],[50]. Our finding that Msx2 is downregulated by Ambn is supported by earlier studies reporting upregulation of Msx2 in ameloblasts from *Ambn* deficient mice [19] and downregulation of MSX2 in human ameloblastoma cells overexpressing AMBN [26]. Notably, *Msx2* deficient mice featured a patency of the posterior frontal suture [32], a phenotype resembling the *Ambn* overexpressor phenotype described in the present study. Underscoring the severe clinical impact of defects caused by changes in Msx levels and suture development, *Msx1/2* double mutant embryos were lacking frontal bones and died at E17 to E18 [51]. Together, these studies indicate that changes in Ambn affect craniofacial growth and suture closure via Msx2.

Evidence from previous studies suggests that the effects of Ambn on Msx2 suppression and calvarial osteoblast proliferation inhibition may be related, as it has been shown that Msx2 increases the expression of CcnD1, inhibits cell differentiation [52], and influences the number of proliferative osteogenic cells in growth centers of the developing mouse skull [53]. Others have reported that calvarial osteoblasts derived from *Msx2* deficient mice had a lower rate of proliferation and demonstrated accelerated osteoblastic differentiation at a later stage when compared to osteoblasts derived from wildtype mice [54]. Our finding that suture mesenchymal cells from transgenic mice reduced CcnD1 and osteogenic gene expression under sub-confluent condition supported a potential connection between the role of Ambn in osteogenic differentiation and its role in cell proliferation inhibition. Together, our data indicate that Ambn affects calvarial osteoblast proliferation and differentiation through suppression of the transcription factor Msx2.

## Conclusions

These data suggested that the extracellular matrix protein Ambn plays a crucial role in cranial suture closure. The effect of Ambn on suture closure may be explained by the inhibitory effect of Ambn on the transcription factor Msx2 and its effect on cell proliferation, resulting in reduced growth and differentiation of calvarial osteoblasts as well as delayed suture closure in mice.
